# Cohort profile: a nationwide study in Dutch CHEK2 c.1100delC families using the infrastructure of the HEreditary Breast and Ovarian cancer study Netherlands – Hebon-CHEK2

**DOI:** 10.1136/bmjopen-2024-086688

**Published:** 2024-10-09

**Authors:** Maartje A.C. Schreurs, Muriel A Adank, Antoinette Hollestelle, Rosa de Groot, Denise J Stommel-Jenner, Christi J. Van Asperen, Margreet G.E.M. Ausems, Lieke P V Berger, Marinus J Blok, Klaartje van Engelen, Frans B L Hogervorst, Willemina Geurts-Giele, Johan J P Gille, Marijke R Wevers, Marjanka K Schmidt, Maartje J. Hooning

**Affiliations:** 1 Erasmus MC Cancer Centre, Rotterdam, The Netherlands; 2 Netherlands Cancer Institute, Amsterdam, The Netherlands; 3 Erasmus Medical Center, Rotterdam, The Netherlands; 4 Leiden University Medical Center, Leiden, The Netherlands; 5 Medical Genetics, UMC, Utrecht, The Netherlands; 6 UMCG, Groningen, The Netherlands; 7 Maastricht UMC+, Maastricht, The Netherlands; 8 Amsterdam UMC Locatie AMC, Amsterdam, Netherlands; 9 Erasmus MC, Rotterdam, The Netherlands; 10 Radboudumc, Nijmegen, The Netherlands; 11 Epidemiology, Netherlands Cancer Institute, Amsterdam, The Netherlands

**Keywords:** Breast tumours, Cancer genetics, Epidemiology

## Abstract

**Abstract:**

**Purpose:**

*CHEK2* c.1100delC is associated with an increased breast cancer risk in women. While this variant is prevalent in the Netherlands (1% in the general population), knowledge of aetiology and prognosis of breast cancer and other tumours in *CHEK2* c.1100delC carriers is lacking. The nationwide HEreditary Breast and Ovarian cancer study the Netherlands (Hebon) cohort aims to answer study questions in families with an increased risk of breast cancer and ovarian cancer. While initially focusing on *BRCA1/2*-variant families, Hebon gradually expanded to include pathogenic variants in other genes associated with breast and/or ovarian cancer over time. This provides an excellent setting to establish a cohort to ultimately study the impact of *CHEK2* c.1100delC on cancer risk prediction and surveillance, breast cancer treatment and prognosis.

**Participants:**

We invited all heterozygous and homozygous *CHEK2* c.1100delC indexes and tested female relatives. 1802 women were included, of whom 1374 were heterozygotes and 938 were breast cancer cases. Pedigrees were collected from all clinical genetic departments. Furthermore, participants completed a detailed questionnaire on hormonal and lifestyle factors, family history, cancer diagnosis and treatment.

**Findings to date:**

Mean age at study inclusion was 53 years. Linkage with the Netherlands Cancer Registry showed a younger age at diagnosis in homozygotes (mean age 41.7 years) and heterozygotes (47.9 years) than non-carriers (51.2 years). Furthermore, carriers were more often diagnosed with grade 2, oestrogen receptor-positive breast cancer and more often developed contralateral breast cancer than non-carriers. Most women consumed alcohol regularly and about half never smoked.

**Future plans:**

Further data linkages with the Netherlands Cancer Registry will allow prospective follow-up and breast cancer risk assessment in unaffected women at the time of genetic testing, risk of contralateral breast cancer and survival in patients with breast cancer. Also, linkage with the nationwide network and registry of histopathology and cytopathology in The Netherlands (PALGA) allows us to retrieve tumour samples to study tumourigenesis.

**STRENGTHS AND LIMITATIONS OF THIS STUDY**

- As part of the ongoing nationwide Hebon cohort established in 1997, thus far containing data from over 50 000 members of families with an increased risk of breast cancer and/or ovarian cancer, the present study includes the largest established *CHEK2* c.1100delC family cohort. This cohort involves 1802 tested female relatives.
- The Hebon-CHEK2 study is a retrospective cohort study, with prospective data collection. Over time, regular data updates from the Netherlands Cancer Registry and the nationwide network and registry of histopathology and cytopathology in The Netherlands (PALGA), enable us to prospectively follow these families over time. Also, follow-up questionnaires will be sent out, establishing the changes in risk factors over time.
- At the moment, the cohort does not contain sufficient follow-up time from unaffected women to assess prospectively age-specific breast cancer risks in our population. This will be needed for a more personalised preventive risk strategy. However, with the planned continuous (prospective) inclusion, this cohort will continue to grow.

## Introduction

 Breast cancer is the most common malignancy in European women.^1^ In the Netherlands, one in seven women will be diagnosed with breast cancer during life, which corresponds with approximately 15 600 new cases in 2022.^2^ It is estimated that 5%–10% of breast cancer cases are caused by disease-associated genetic variants.^3^

The nationwide Hereditary Breast and Ovarian cancer study the Netherlands (Hebon) was established in 1997. Hebon includes members of families with an increased risk of breast cancer or ovarian cancer. One of the disease-associated genetic variants that was more recently implemented in diagnostic testing in the Netherlands is the *CHEK2* c.1100delC pathogenic variant, which is most commonly found in Northern European countries. Studies have shown that approximately 1% of the Dutch population carries this variant,^4^ and up to 5% of breast cancer families.^5^ *CHEK2* c.1100delC results in malfunction of the checkpoint kinase 2, which is a multifunctional enzyme with essential involvement in the induction of cell cycle arrest.^6^ It is activated in the presence of DNA damage,^7^ and therefore, plays an important role in tumour suppression. As a result, women with the *CHEK2* c.1100delC variant have a moderately increased risk of breast cancer, which further increases with a positive family history of breast cancer.^8 9^ Therefore, diagnostic testing for the c.1100delC variant was initiated in the Netherlands in 2014. All first-degree female relatives of the index were eligible for genetic testing. From 2018 onwards, also second-degree female relatives became eligible (modified cascade testing).^10^ Depending on their family history of breast cancer, women from *CHEK2* families who tested negative themselves might still be eligible for additional breast cancer surveillance.^8^

When diagnosed with breast cancer, their age at diagnosis is younger than those of patients with sporadic breast cancer.^89 11^,^16^ The majority of breast tumours from *CHEK2* c.1100delC heterozygotes are oestrogen receptor (ER) positive.^417^,^19^ Furthermore, after the primary breast cancer diagnosis, carriers (both heterozygous and homozygous) more often develop contralateral breast cancer^1620^,^22^ and tend to have worse survival than non-*CHEK2* carriers.^16^,^1823^ Previous studies were primarily conducted on retrospectively collected breast cancer cases, that were tested in a research setting. However, to study the yet-unanswered research questions, such as risks of cancer other than the breast, response to cancer treatment and survival after breast cancer diagnosis, systemic prospective data collection is needed.^24^

With this cohort, we ultimately aim to study the impact of *CHEK2* c.1100delC on cancer risk prediction and surveillance, and treatment of breast cancer and prognosis within *CHEK2* c.1100delC families. The high prevalence of this variant in the Netherlands and the availability of the Hebon infrastructure provide a unique opportunity to study this in a more homogeneous setting (Hebon-CHEK2 study). In the current study, we describe the retrospective collection of female relatives who were tested for the familial *CHEK2* c.1100delC variant. Furthermore, linking this cohort with national databases, such as the Netherlands Cancer Registry and with the nationwide network and registry of histopathology and cytopathology in The Netherlands (PALGA), enabled us to retrieve harmonised data on cancer diagnosis, tumour characteristics and treatment. In addition, it will enable us to prospectively study (contralateral) breast cancer risk and prognosis in the future.

## Cohort description

### Hebon study

The nationwide Hereditary Breast and Ovarian cancer study, the Netherlands (Hebon study) is a collaboration between all clinical genetic departments in the Netherlands (from all eight university medical hospitals and the Netherlands Cancer Institute). In 1997, the predecessor of the Hebon study (GEO-Hebon) was initiated,^25^ primarily including members of families with a (detected) pathogenic disease-associated *BRCA1* and/or *BRCA2* variants.

As part of the Hebon-CHEK2 study, we invited (1) all index cases who were eligible for diagnostic testing and tested positive for this *CHEK2* variant and (2) all female relatives who were tested for the familial *CHEK2* c.1100delC variant, independent of their own *CHEK2* status. Therefore, all diagnostically confirmed *CHEK2* families were retrospectively selected between 1 January 2019 and 1 July 2021 (exact dates vary between centres).

As part of this study, we updated the Hebon questionnaire, making it suitable for families carrying (likely) pathogenic variants in genes other than *BRCA1* and *BRCA2*, as currently everyone who had breast and/or ovarian cancer genetic panel testing, regardless of their own test result, is eligible to participate. Some questions, such as ovarian cancer risk and preventative risk-reducing strategies, are not applicable to all other genetic variants and are, therefore, not the main focus of the questionnaire anymore. Also, we made some changes in the topics (eg, ovarian cancer risk is not as primary focus) that are addressed in the questionnaire and updated the questions for current use.

### Patient and public involvement

Patient and public involvement was embedded in the Hebon study by involving the Hebon panel, in which patients with hereditary breast cancer and/or ovarian cancer participated. They reviewed and provided feedback on the protocol, study design and questionnaire. Results are disseminated via the Hebon website. Also, patients and the public are welcome to visit the annual Hebon congress, where the latest results regarding hereditary breast and/or ovarian cancer are shared with colleagues.

### Selection, invitation and response

First, we retrospectively selected all *CHEK2* c.1100delC positive families from each diagnostic laboratory (n=2568). We verified the patients’ records, to ensure that women did not object to their data being used for research purposes. After that, we checked the variant status and whether women were counselled for their genetic status. As the selection was based on *CHEK2* c.1100delC status only, it might be possible that women from families with other pathogenic variants in addition to the *CHEK2* c.1100delC are included in this study. However, when this information was available, these women were excluded. Overall, the likelihood of compound *CHEK2* heterozygosity or the combination of *CHEK2* c.1100delC and other pathogenic variants in one of the breast cancer-associated genes is small.

In total, 198 women who were initially included as being from non-*BRCA1/2* families were later found to be from *CHEK2* c.1100delC families. Therefore, they were directly included in this study (without receiving a new invitation) because they already participated in Hebon. Some centres chose to exclude women with psychological problems, difficulties with understanding the Dutch language, or palliative stage of breast cancer. In these situations, receiving an invitation could do more harm than good (n=54).

After linking the remaining (n=2316) eligible women with the Dutch Personal Records Database (BRP), from which their current address and vital status were retracted, invitations were sent out to those alive and who resided in the Netherlands. After 4–6 weeks, women who had not responded, received a reminder letter. A maximum of two reminders were sent to women who had not responded, after which they were marked as non-responders and only limited, pseudonymised data linkage was possible. Also, women who were deceased before sending out invitations were included, as the exclusion of these women would probably likely introduce bias (paragraph 5.4.2.b. of the Dutch Code of Conduct for Health Research).

**Figure 1:**
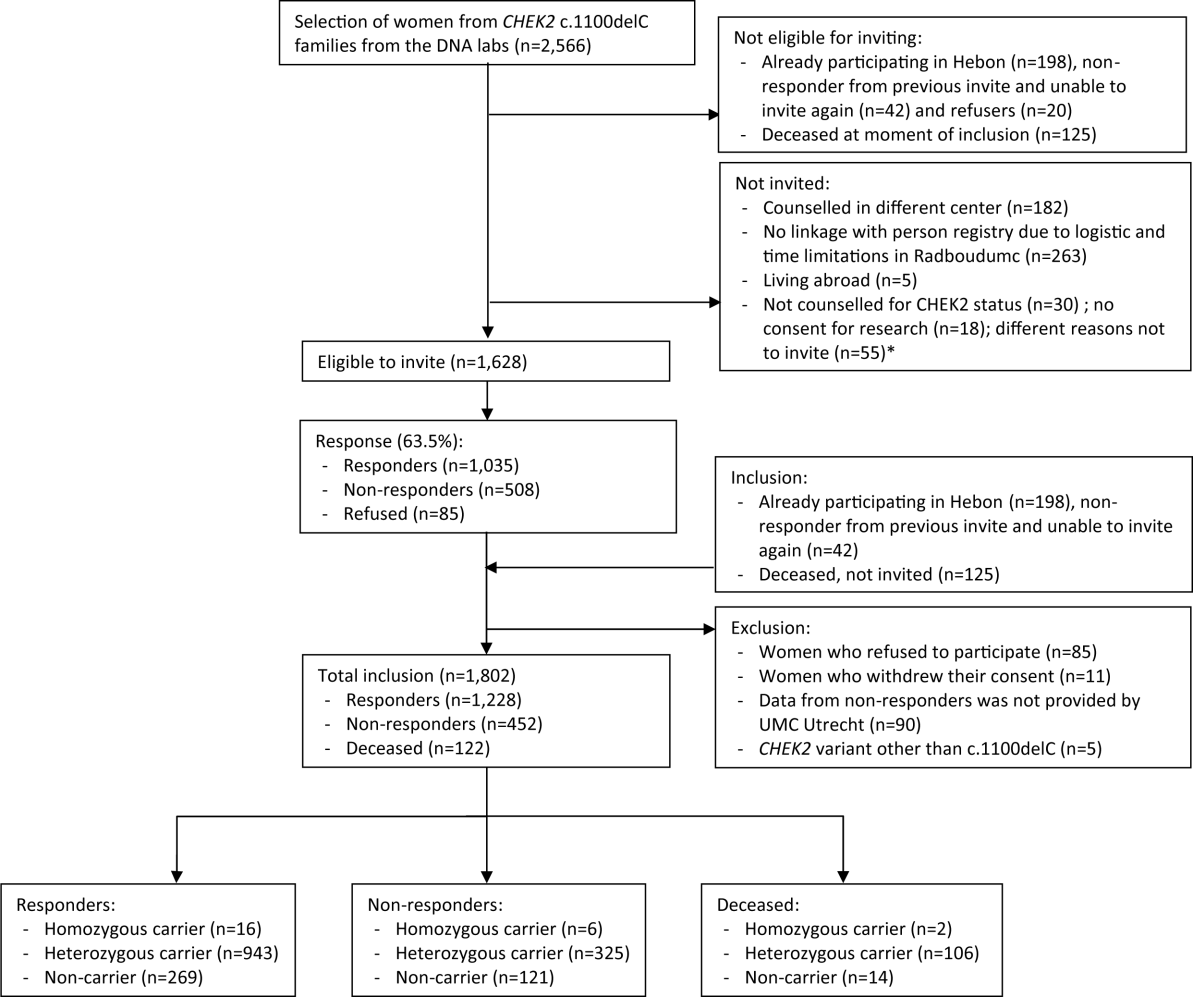
Flow chart of women included in this study. Non-responders and deceased women were also included in this study, however, only limited data collection for these women was possible. *Reasons not to invite were based on clinical geneticists’ input and included psychological problems, not understanding the Dutch language well enough, or when women were in the palliative phase of disease (and) situations in which receiving an invitation could cause more harm than good. UMC=University Medical Center.

In total, we invited 1630 women to participate in this study, of whom 1035 women responded and signed informed consent (response rate of 63.5%) (figure 1). A total of 198 women already participated in Hebon before and were, therefore, not approached again. After the invitation rounds, we included 1228 responders, 452 non-responders and 122 deceased women. All women received a unique Hebon research number as part of their inclusion. Identifying data of individuals is only accessible to the Hebon study coordinators and was not provided in further data analyses. When requesting data from the Netherlands Cancer Registry or PALGA, study coordinators will use the individual data to ensure correct linkage.

Currently, Hebon and all Dutch Clinical Genetic centres are setting up a streamlined system to invite individuals who had breast and/or ovarian cancer genetic panel testing (ie, regardless of test outcome results and/or being affected with breast and/or ovarian cancer) and counselled by a clinical geneticist to semiautomatically be invited within 6 months after testing. This prospective approach will gradually increase the number of tested relatives from *CHEK2* c.1100delC families within the Hebon study.

### Data collection

Available data from our Hebon-CHEK2 study population are described in table 1. For the Hebon-CHEK2 study, we currently have collected data from three main sources: (1) data derived from clinical genetic departments and medical files, including variant status, surveillance advice and pedigrees as part of the selection process; (2) the self-reported Hebon risk factor questionnaire, including detailed questions on height and weight at time of completing the questionnaire, pregnancies, hormonal contraceptive use, tubal ligation, (peri)menopausal status, breast surveillance, cancer diagnosis, smoking and alcohol consumption which was obtained (table 2) and (3) data from the Netherlands Cancer Registry (also including pathology data from PALGA), including data on age and year of breast cancer diagnosis, tumour characteristics, treatment and contralateral breast cancer occurrence (table 3).

**Table 1:** Overview of available data for the *CHEK2* c.1100delC female study population

Data source	Available info	Responders	Non-responders	Deceased	Overview
Hebon (online) self-reported questionnaire*	Ethnicity, height and weight, parity, hormonal contraceptive use, menarche, (peri) menopause, genetic tests, conditions (including myoma, PCOS, heart conditions, neurological conditions), types of examinations (including X-ray), breast screening, cancer diagnosis and treatment, smoking, alcohol consumption, physical activity and family history of cancer.	Available	Not applicable	Not applicable	table 2
Clinical information; specifically breast cancer diagnosis and follow-up	Linkage with Netherlands Cancer Registry, providing information on age and year of breast cancer diagnosis, tumour characteristics, treatment and vital status.	Available	Limited pseudonymised information available.	Available	table 3
	Documented by the Netherlands Cancer Registry, providing more detailed information on surveillance, radiation therapy, recurrent disease, metastatic disease, treatment for recurrent and metastatic disease and follow-up	Collected	–	Collected
Pedigrees	Pedigrees collected as used during counselling at the clinical genetic centres. Contains information on family structures, genetic testing, cancer diagnoses (including year or age, type of cancer, validation of tumour) and vital status.	Collected; the availability and detail in pedigrees vary between centres	Pedigrees of responders were collected. As this is a familial study, it is possible that non-responders or deceased were also included in these pedigrees. However, we did not specifically collect pedigrees for these groups.	

* pPaper questionnaires were available to participants onon request.

PCOSpolycystic ovarian syndrome

**Table 2:** Overview of available information from the Hebon self-reported questionnaire for *CHEK2* c.1100delC variant carriers and non-carriers of familial *CHEK2* c.1100delC

	Non-carriersn=246	Heterozygotesn=886	Homozygotesn=15	P value
Age in years at time of questionnaire completion, mean (SD) min–max	52.3 (12.5)26–84	53.7 (12.5)23–88	50.5 (12.5)32–74	0.19
Country of birth, n (%)				0.91
The Netherlands	211 (98.6)	745 (98.7)	13 (100.0)	
Other	3 (1.4)	10 (1.3)	–	
Missing, n	32	131	2	
Height and weight				
Current weight (kg), mean (SD)	76 (14)	75 (14)	72 (10)	0.40
Missing, n	24	84	1	
Current height (cm), mean (SD)	170 (7)	170 (6)	168 (6)	0.44
Missing, n	22	65	0	
Fertility				
Past or current pregnancy, n (%)				0.18
No	43 (17.8)	118 (13.5)	3 (21.4)	
Yes	198 (82.2)	756 (86.5)	11 (78.6)	
Missing, n	5	12	1	
Oral contraceptive use, n (%)				0.98
No	145 (61.7)	527 (61.5)	9 (64.3)	
Yes	90 (38.3)	330 (38.5)	5 (35.7)	
Missing, n	11	29	1	
Tubal ligation, n (%)				0.01
No	216 (90.4)	745 (86.8)	9 (64.3)	
Yes	23 (9.6)	113 (13.2)	5 (35.7)	
Missing, n	7	28	1	
(Peri)menopausal, n (%)				0.12
No	143 (60.3)	573 (67.2)	8 (57.1)	
Yes	94 (39.7)	280 (32.8)	6 (42.9)	
Missing, n	9	33	1	
Breast cancer surveillance				
Screened via mammography, n (%)				<0.001
No	32 (14.0)	52 (6.2)	–	
Yes	197 (86.0)	781 (93.8)	14 (100.0)	
Missing, n	17	1	1	
Screened via MRI, n (%)				<0.001
No	188 (82.8)	589 (70.5)	7 (50.0)	
Yes	39 (17.2)	246 (29.5)	7 (50.0)	
Missing, n	19	51	1	
Cancer diagnosis, self-reported				
All cancer diagnoses, n (%)				<0.001
No	162 (70.1)	294 (35.1)	–	
Yes	69 (29.9)	543 (64.9)	14 (100.0)	
Missing, n	15	49	1	
Breast cancer diagnosis of all cancer diagnoses, n (%)				0.001
No	11 (15.9)	25 (4.6)	1 (7.1)	
Yes	58 (84.1)	518 (95.4)	13 (92.9)	
No cancer reported, n	169	294	0	
Missing, n	15	49	1	
Lifestyle factors				
Smoking, n (%)				0.11
Never	126 (54.3)	416 (49.9)	6 (42.9)	
Ever	82 (35.3)	358 (42.9)	8 (57.1)	
Current	24 (10.3)	60 (7.2)	–	
Missing, n	14	52	1	
Alcohol consumption, n (%)				0.14
Never	110 (47.8)	355 (43.0)	3 (23.0)	
Ever	120 (52.2)	470 (57.0)	10 (77.0)	
Missing, n	16	61	2	

Percentages may not add up to 100% due to rounding. Questionnaire data are only available for responders of this study. In total, we obtained self-reported data from 1147 out of the 1228 responders (93.4% started the questionnaire after informed consent).

**Table 3:** Overview of data on breast cancer occurrence, tumour characteristics and treatment as provided by the Netherlands Cancer Registry

	Non-carriersN=64	HeterozygotesN=856	HomozygotesN=18	P value
Responders, n (%)	48 (75.0)	604 (70.6)	12 (66.7)	
Deceased, n (%)	4 (6.3)	61 (7.1)	1 (5.6)	
Non-responders, n (%)	12 (18.8)	191 (22.3)	5 (27.8)	
**Patientcharacteristics**				
Age at first breast cancer diagnosis, mean (SD)	51.2 (10.5)	47.9 (10.6)	41.7 (12.6)	0.002
Min–max	27–80	22–84	24–70	
Age category, n (%)				0.004
<40	7 (10.9)	188 (22.0)	9 (50.0)	
40–50	24 (37.5)	347 (40.5)	4 (22.2)	
>50	33 (51.6)	321 (37.5)	5 (27.8)	
Year at first breast cancer diagnosis, median	2011	2014	2015	0.15
Min–max	1993–2019	1976–2021	1993–2019	
Year category, n (%)				0.05
<2000	8 (12.5)	82 (9.6)	3 (16.7)	
2000–2005	13 (20.3)	87 (10.2)	1 (5.6)	
2005–2010	8 (12.5)	106 (12.4)	3 (16.7)	
2010–2015	18 (28.1)	208 (24.3)	1 (5.6)	
≥2015	17 (26.6)	373 (43.6)	10 (55.6)	
**Follow-up**				
Follow-up time in years, median	10.5	7.4	6.7	0.06
Min–max	2.2–28.9	0.1–46.1	2.9–28.8	
Contralateral breast cancer, n (%)				
Total incidences	10 (15.6)	207 (24.2)	6 (33.3)	0.19
Within first 5 years	5 (7.8)	104 (12.2)	3 (16.7)	0.48
Within first 10 years	6 (9.4)	142 (16.6)	3 (16.7)	0.32
Within first 15 years	9 (14.1)	174 (20.3)	4 (22.2)	0.47
**Tumourcharacteristics**				
Morphology, n (%)				0.002
Ductal	43 (67.2)	692 (80.8)	12 (66.7)	
Lobular	7 (10.9)	53 (6.2)	2 (11.1)	
Medullary	–	3 (0.4)	1 (5.6)	
Mixed (ductal and lobular)	2 (3.1)	36 (4.2)	–	
Mucinous	3 (4.7)	10 (1.2)	–	
Tubular	1 (1.6)	6 (0.7)	1 (5.6)	
Other	8 (12.5)	56 (6.5)	2 (11.1)	
Behaviour, n (%)				0.65
In situ	8 (12.5)	84 (9.8)	1 (5.6)	
Invasive	56 (87.5)	772 (90.2)	17 (94.4)	
Size, n (%)				0.12
≤2 cm	35 (62.5)	536 (66.6)	10 (55.6)	
>2 and ≤5 cm	18 (32.1)	214 (26.6)	4 (22.2)	
>5 cm	3 (5.4)	55 (6.8)	4 (22.2)	
Missing, n	8	51	0	
Nodal status, n (%)				0.35
Negative	40 (63.5)	503 (59.7)	8 (44.4)	
Positive	23 (36.5)	339 (40.3)	10 (55.6)	
Missing, n	1	14	0	
Grade, n (%)				0.06
1	12 (22.2)	130 (18.1)	–	
2	20 (37.0)	369 (51.3)	7 (46.7)	
3	22 (40.7)	221 (30.7)	8 (53.3)	
Missing, n	10	136	3	
Multifocality, n (%)				0.85
No	40 (80.0)	577 (77.4)	13 (81.3)	
Yes	10 (20.0)	169 (22.7)	3 (18.8)	
Missing, n	14	110	2	
ER status, n (%)				<0.001
Negative	11 (25.6)	44 (6.7)	4 (23.5)	
Positive	32 (74.4)	612 (93.3)	13 (76.5)	
Missing, n	21	200	1	
PR-status, n (%)				0.06
Negative	14 (33.3)	139 (21.2)	6 (37.5)	
Positive	28 (66.7)	517 (78.8)	10 (62.5)	
Missing, n	22	200	2	
HER2 status, n (%)*				0.001
Negative	31 (81.6)	470 (75.8)	4 (28.6)	
Equivocal	–	6 (1.0)	–	
Positive	7 (18.4)	144 (23.2)	10 (71.4)	
Missing, n	26	236	4	
**Treatment**				
Surgery, n (%)				0.55
No surgery	3 (4.7)	19 (2.2)	1 (5.6)	
Lumpectomy	35 (54.7)	444 (51.9)	7 (38.9)	
Mastectomy	26 (40.6)	379 (44.3)	10 (55.6)	
Other/type unknown	–	14 (1.6)	–	
Radiotherapy, n (%)				0.12
No	37 (57.1)	402 (47.0)	6 (33.3)	
Yes	27 (42.9)	454 (53.0)	12 (66.7)	
Chemotherapy, n (%)				0.07
No	34 (53.1)	391 (45.7)	4 (22.2)	
Yes	30 (46.9)	465 (54.3)	14 (77.8)	
Endocrine therapy, n (%)				0.16
No	34 (53.1)	350 (40.9)	7 (38.9)	
Yes	30 (46.9)	506 (59.1)	11 (61.1)	
Targeted therapy, n (%)				<0.001
No	57 (89.1)	721 (84.2)	9 (50.0)	
Yes	7 (10.9)	135 (15.8)	9 (50.0)	

* HER2 status has been determined since 2000, which also partly explains the number of missings in this variable. Metachronous contralateral breast cancer risk is defined as breast cancer in other breast at least 3 months after primary breast cancer diagnosis. Percentages may not add up to 100% due to rounding. Numbers in this table are derived from the Netherlands Cancer Registry, and therefore, differs from the total number of included women and the self-reported BC data. For 17 responders (2 non-carriers and 15 heterozygotes), 2 BC diagnoses occurred on the same date. For the non-responders, the exact date was unknown. However, for 12 non-responders (1 non-carriers, 10 heterozygotes, 1 homozygote), 2 BC diagnoses occurred in the same month. In those cases, the tumour with the highest stage was used.

ER, oestrogen receptor; HER2, human epidermal growth factor receptor 2; PR, progesterone receptor

Statistical analyses were performed using STATA (V.18.0). Descriptive statistics are showed as mean and SD or median and a range. We used Pearson’s χ^2^ test for categorical data and ANOVA test for continuous data to calculate differences in patients’ characteristics.

### Cohort description and missing data

In total, we have 1802 women included in this study; 24 homozygotes, 1374 heterozygotes and 404 non-carriers. All participants were asked to complete the Hebon questionnaire. In total, 1147 women (93.4% of the 1228 responders) fulfilled this request, of whom the majority were between 2019 and 2021. A small group of women already participating in Hebon earlier completed the questionnaire between 2012 and 2016. Although we nudged participants to answer the complete online questionnaire, we observed that the number of missing data varied between topics (table 2). For example, for height and weight (7.8% missings in height and 9.5% missings in weight), the number of missing data was higher than for pregnancies (1.6%).

Linkage with the Netherlands Cancer Registry resulted in data on breast cancer occurrence for 938 women, of whom 856 heterozygotes (62.3% of the total of 1374 heterozygotes), 18 homozygotes (75.0% of the total of 24 homozygotes) and 64 non-carriers (15.8% of the total of 404 non-carriers). The number of missing data for the tumour characteristics is limited, ranging from 1.6% (n=15) for nodal status to 28.4% (n=266) for Human epidermal growth factor receptor 2 (HER2) status.

## Findings to date

This cohort includes the largest homozygous *CHEK2* c.1100delC population known to date.^4 26 27^ Furthermore, by also including women who tested negative for this variant, we obtained a control group that is comparable with regard to family history and has a similar genetic background. Furthermore, we obtained pedigrees from 794 out of the 811 responding families (97.9%), including data on cancer occurrence in addition to breast.

In total, we obtained self-reported data from 1147 out of the 1228 responders (93.4% started the questionnaire after informed consent). Data showed that almost all women were born in the Netherlands. The mean age at time of inclusion was 53 years (ranging from 23 to 88 years). The majority of these women have children, and about 60% of the women were (peri)menopausal at the time of completing the questionnaire. Self-reported breast surveillance (both via mammography and MRI) was significantly more often conducted in carriers than in non-carriers. Significantly more carriers than non-carriers are diagnosed with breast cancer and other types of cancer. In total, 626 women were reported to have cancer, of whom 589 had breast cancer (94.1%). About half of the population never smoked, and most of the women consumed more than one alcoholic beverage a week for at least 1 year in their lives (table 2).

Linkage with the Netherlands Cancer Registry showed that 938 women from our cohort were diagnosed with breast cancer, of whom, 664 were responders. This number is higher than expected from the questionnaire, as part of the women did not start or complete the whole questionnaire. Mean age at breast cancer diagnosis was significantly lower for *CHEK2* c.1100delC carriers than non-carriers (47.9±10.6 years for heterozygous carriers, 41.7±12.6 years for homozygous *CHEK2* carriers and 51.2±10.5 years for non-carriers), which is in line with previous studies.^48 9 11^,^16 28^ The median year of breast cancer diagnosis was 2014. The median follow-up time from breast cancer occurrence onwards is 7.5 years (range 0.1–46.1 years). As expected, carriers more often were diagnosed with ER-positive breast cancer.^416^,^20^ In addition, carriers were significantly more often HER2-positive than non-carriers. Although not confirmed in larger settings, this association has been described in a small study.^29^ Finally, no significant differences in treatment were found, except for targeted therapy, which is more often given in carriers (table 3).

Using pedigree information from 609 *CHEK2* c.1100delC families, we studied whether families are at risk of other cancers in addition to the breast.^30^ In this study, we found an increased risk of colorectal cancer and haematological cancers for women from *CHEK2* c.1100delC families. Even though there seems to be an underreporting of cancer in the male relatives in this study, we also found an increased risk of breast cancer and colorectal cancer in men.

## Strengths and limitations

This cohort has several strengths. It is part of an ongoing nationwide cohort established in 1997, which includes data from over 50 000 members of families with an increased risk of breast cancer and/or ovarian cancer, who were tested primarily for *BRCA1* and *BRCA2* genetic variants. Since 2014 routine testing in the Netherlands also includes the *CHEK2* c.1100delC (pathogenic) variant, while in recent years the test panel has been extended with the complete *CHEK2* gene, *PALB2*, *ATM*, *RAD51C, RAD51D*, *BRIP1* and *BARD1* genes. To date, the Hebon study includes the largest *CHEK2* c.1100delC family cohort, including 1802 female tested relatives which is the focus for this manuscript. These data provide a unique opportunity to study the impact of this *CHEK2* variant on cancer occurrence, including breast cancer and contralateral breast cancer, and survival in a homogeneous setting. The Hebon-CHEK2 study is a retrospective cohort study, with prospective data collection. Over time, regular data updates from the Netherlands Cancer Registry (including pathology data from PALGA) enable us to prospectively follow these families. This provides us with updated information on breast cancer incidence as well as other cancers and characteristics and also on survival of patients with breast cancer. Detailed information on risk factors (including body mass index (BMI), hormonal factors, smoking, alcohol consumption, family history, systemic therapy, radiation and surveillance) is available from self-reported questionnaires. Also, follow-up questionnaires are sent out, establishing the changes in risk factors over time. Using this information, we can study the association between risk factors such as BMI, parity or hormonal contraceptive use and cancer occurrence, including breast cancer and contralateral breast cancer, and the effect of type and duration of systemic therapy on survival.

While we have the largest cohort profile on CHEK2 families, this cohort has some limitations. At present, the cohort does not contain sufficient data from unaffected women to assess age-specific breast cancer risks in our population. This will be needed for a better personalised preventive risk strategy. However, with the recent startup of the continuous (prospective) inclusion, this cohort will continue to grow.

## Future plans

Using the currently available data, we aim to study cancer risks in our heterozygous and homozygous *CHEK2* c.1100delC families. Using this information, we could create awareness of other cancer risks and potentially offer additional surveillance for other types of cancer than breast cancer, resulting in earlier detection and better survival.

With this prospective cohort study, we will be able to collect more data over time. For example, we may collect updated pedigrees from clinical genetic departments to study changes in cancer family history (pathologically confirmed) new cancer diagnoses or the uptake of genetic testing within families. Also, every 5–10 years follow-up questionnaires will be sent out, providing more up-to-date information on family history and hormonal (eg, oral contraceptive use, hormone replacement therapy, parity), lifestyle (eg, alcohol use, smoking) risk factors and preventive breast surgeries but also enabling us to study changes in risk factors (such as weight or hormonal contraceptive use) and its effect on cancer occurrence or survival. In addition, mammographic imaging and radiology reports will be used to study the detectability of breast cancer on mammographic images. Furthermore, we could invite responders to participate in additional research, asking them for a blood sample or filling out a questionnaire on a specific subject. Also, we may collect tumour samples through a central PALGA request for further molecular analysis on tumourigenesis. Finally, future linkages with the Netherlands Cancer Registry will take place. This will result in longer follow-up time for known breast cancer cases and identify new patients with breast cancer increasing power to prospectively assess (contralateral) breast cancer risk, also allowing for further stratification based on ER-status and menopausal status. Furthermore, this would enable us to study prognosis after breast cancer occurrence in the least biased way.

In addition, we aim to expand the cohort to include tested males and tested relatives from families where other (likely) pathogenic *CHEK2* variants are found. Including these families in this cohort would allow us to study risks for these families as well and compare them with *CHEK2* c.1100delC families.

## Data Availability

Data are available on reasonable request.
