# Supplementary material for: Cohort profile: a nationwide study in Dutch CHEK2 c.1100delC families using the infrastructure of the HEreditary Breast and Ovarian cancer study Netherlands – Hebon-CHEK2
Source: BMJ Open. 2024 Oct 9;14(10):e086688. doi: 10.1136/bmjopen-2024-086688 (PMC11474769; doi:10.1136/bmjopen-2024-086688)
Supplement: Supplementary file 1 [file bmjopen-2024-086688.reviewer_comments.pdf]

PEER REVIEW HISTORY

BMJ Open publishes all reviews undertaken for accepted manuscripts. Reviewers are asked to complete a checklist review form and are provided with free text boxes to elaborate on their assessment. These free text comments are reproduced below.

ARTICLE DETAILS

Title (Provisional)

Cohort profile: a nationwide study in Dutch CHEK2 c.1100delC families using the infrastructure of the HEreditary Breast and Ovarian cancer study Netherlands: Hebon-CHEK2.

Authors

Schreurs, Maartje; Adank, Muriel A.; Hollestelle, Antoinette; de Groot, Rosa; Stommel-Jenner, Denise J; Van Asperen , Christi; Ausems, Margreet; Berger, Lieke P.V.; Blok, Marinus J.; van Engelen, Klaartje; Hogervorst, Frans B.L.; Geurts-Giele, Willemina; Gille, Johan JP; Wevers, Marijke R; Schmidt, Marjanka K; Hooning, M. J.

VERSION 1 - REVIEW

**Reviewer**	**1**
**Name**	**Whitaker , Kristen**
**Affiliation**	**MedStar Health**
**Date**	**13-May-2024**
**COI**	**N/A**

In this manuscript, the authors describe the clinicopathological features of breast cancers diagnosed in carriers of PV in CHEK2 c.1100delC mutations, as well as describe the impact of these mutations on survival, contralateral breast cancer risk, and treatment. Although, the others note that their aim is studying the impact of CHEK2

32 c.1100delC on cancer risk prediction and surveillance, breast cancer treatment and prognosis, the manuscript is ultimately is descriptive in nature and does not address the impact on surveillance in neither carriers or noncarriers. My suggestion would be for modification of the described study objectives to more accurately reflect what is being presented in the manuscript-- Perhaps, the authors could consider altering the objectives to describe the clinicopathological features of breast cancer in carriers of CHEK2 c.1100delC PV, as well as describe the risk of contralateral breast cancer risk, survival, and breast cancer treatment among these individuals. The biggest weakness of the manuscript is that several results are presented without any statistical plan discussed. For example, higher risks of contralateral breast cancer, ER breast cancer, HER2 breast cancer, among CHEK2 mutation

carriers compared to non-carriers. It is unclear to me what statistical methods were used and if these results were in fact statistically significant. Additionally, this study and manuscript will contribute most meaningfully to the scientific community if the results presented aid in the understanding of both patients and physicians about the risks of contralateral breast cancer in these mutation carriers so that this information can be used to help patients make more tailored decisions about the benefit of contralateral prophylactic mastectomy once diagnosed with a breast cancer. Stating that there is an increased risk of contralateral breast cancer risk, without given specifics with cumulative incidence of contralateral breast cancer risk at 5, 10, 15 years is less helpful. At this point, the follow-up period may not be long enough to provide this data. However, given that the study has a cohort of carriers, and non-carriers, my suggestion would be do present the increase in contralateral breast cancer using Hazard ratios. Presenting a table showing this would be helpful. Additionally, the authors fail to include as reference the most recent and high impact manuscript from the CARRIERS study which examined contralateral breast cancer risk(Yadav S, Boddicker NJ, Na J, Polley EC, Hu C, Hart SN, Gnanaolivu RD, Larson N, Holtegaard S, Huang H, Dunn CA, Teras LR, Patel AV, Lacey JV, Neuhausen SL, Martinez E, Haiman C, Chen F, Ruddy KJ, Olson JE, John EM, Kurian AW, Sandler DP, O'Brien KM, Taylor JA, Weinberg CR, Anton-Culver H, Ziogas A, Zirpoli G, Goldgar DE, Palmer JR, Domchek SM, Weitzel JN, Nathanson KL, Kraft P, Couch FJ. Contralateral Breast Cancer Risk Among Carriers of Germline Pathogenic Variants in ATM, BRCA1, BRCA2, CHEK2, and PALB2. J Clin Oncol. 2023 Mar 20;41(9):1703-1713. doi: 10.1200/JCO.22.01239. Epub 2023 Jan 9. PMID: 36623243; PMCID: PMC10022863) I would suggest the authors read this manuscript if they have not and also include it as reference to ensure references are up to date. As a point of interest, given that this study has a larger cohort of CHEK2 patients than the CARRIERS study, it would be interesting if they conducted an exploratory analysis examining the risk of contralateral breast cancer in the CHEK2 mutation carriers by ER status and menopausal status as differences in the risk of contralateral breast cancer were found in the CARRIERS study based off these factors. At this point, we still have limited understanding about CHEK2-associated breast cancer, particularly about the risk of contralateral breast cancer in these individuals. This cohort and study can provide valuable knowledge to improve risk prediction and cancer care in CHEK2 mutation carriers for years to come. However, further details related to risks associated with these mutations with regards to contralateral breast cancer will increase the impact of this manuscript to the scientific community. Additionally, as the authors ultimately wish to use this cohort to expand our knowledge potentially of additionally cancer risks, I would encourage them to expand their cohort to include men, especially with emerging data related to elevated prostate cancer risk.

**Reviewer**	**2**
**Name**	**Moscetti , Luca**

Affiliation **Universitaria di Modena, Oncology**

Date **01-Jul-2024**

COI **honoraria from Roche, EISAI, Daichii Sankyo, Novartis, Pfizer, Gilead, Eli Lilly**

Thanks to the authors for their excellent work. I only have two comments:

- Has the population included in the study been tested for mutations other than the CHEK2 c.1100delC variant to exclude a concomitant mutation?

- Table 2 shows the available information on the characteristics of the patients. Was any univariate/multivariate analysis performed to evaluate the role in the association with the CHEK2 mutation? I am aware that some habits are already known as potential risk factors for breast cancer (smoking, alcohol consumption), but it might be interesting to comment on them.

VERSION 1 - AUTHOR RESPONSE

We thank you for the opportunity to revise our manuscript, entitled "Cohort profile: a nationwide study in Dutch CHEK2 c.1100delC families using the infrastructure of the HEreditary Breast and Ovarian cancer study Netherlands: Hebon-CHEK2." (bmjopen-2024-086688).

We appreciate the Reviewers' useful comments on our manuscript. We have revised sections of the manuscript in line with these comments. All questions by the Reviewers (black text) are answered (blue text) on a point-by-point basis with references made to the location of the changes in the track-changes version of the manuscript.

Thank you for your consideration.

RESPONSE TO THE COMMENTS:

Reviewer #1:
In this manuscript, the authors describe the clinicopathological features of breast cancers diagnosed in carriers of PV in CHEK2 c.1100delC mutations, as well as describe the impact of these mutations on survival, contralateral breast cancer risk, and treatment. Although, the others note that their aim is studying the impact of CHEK2 32 c.1100delC on cancer risk prediction and surveillance, breast cancer treatment and prognosis, the manuscript is ultimately is descriptive in nature and does not address the impact on surveillance in neither carriers or noncarriers.

Comment 1: My suggestion would be for modification of the described study objectives to more accurately reflect what is being presented in the manuscript-- Perhaps, the authors could consider altering the objectives to describe the clinicopathological features of breast cancer in carriers of CHEK2 c.1100delC PV, as well as describe the risk of contralateral breast cancer risk, survival, and breast cancer treatment among these individuals.

Response 1: Thank you for your comments. The current objectives are based on the overall aims of the cohort. This cohort profile primarily describes the characteristics of the participants of the cohort, as we are still working on the analyses regarding the main outcomes (including contralateral breast cancer risk and survival). Therefore, this has been clarified in the introduction.

Introduction (page 4, line 96-97):

"~~We~~ With this cohort, we ultimately aim to study the impact of CHEK2 c.1100delC on cancer risk prediction and surveillance, and treatment of breast cancer and prognosis within CHEK2 c.1100delC families."

Comment 2: The biggest weakness of the manuscript is that several results are presented without any statistical plan discussed. For example, higher risks of contralateral breast cancer, ER breast cancer, HER2 breast cancer, among CHEK2 mutation carriers compared to non-carriers. It is unclear to me what statistical methods were used and if these results were in fact statistically significant.

Response 2: We agree that there is no statistical plan included in this manuscript. This is in line with the author guidelines for a cohort profile. However, we have added p-values in the Tables to show if there is a significant difference between the groups regarding self-reported risk factors (Table 2) and the clinicopathological features of breast cancer (Table 3), and added some information on the statistics for that in the cohort description; data collection. Results on statistically significant differences in the findings to date.

Cohort description; data collection (page 7, line 183-185):

"Statistical analyses were performed using STATA (version 18.0). Descriptive statistics are showed as mean and standard deviation (SD) or median and a range. We used Pearson's χ2 test for categorical data and ANOVA test for continuous data to calculate differences in patients' characteristics."

Findings to date (page 8, line 214-217):

"Self-reported breast surveillance (both via mammography and MRI) was more often conducted in carriers than in non-carriers. Significantly more carriers than non-carriers are diagnosed with breast cancer and other types of cancer. In total, ~~638~~ 626 women reported to have cancer, of whom ~~592~~ 589 breast cancer (~~92.8~~ 94.1%)."

Findings to date (page 8, line 227-236):

"~~Contralateral breast cancer was more often found in carriers than in non-carriers, which is in line with previous studies (16, 20, 21). Overall, the majority of women were diagnosed with invasive breast cancer. The majority of the heterozygotes were diagnosed with a ductal morphology, and carriers were more often diagnosed with lymph node positive breast cancer.~~ As expected, carriers significantly more often were diagnosed ~~with grade 2,~~ ER-positive breast cancer (4, 16-20). In addition, carriers

were significantly more often HER2-positive than non-carriers. Although not confirmed in larger settings, this association has been described in a small study (28). Finally, no significant differences in treatment were found, except for targeted therapy, which is more often given in carriers (Table 3). ~~*carriers were more often treated with radiotherapy, chemotherapy, endocrine therapy and targeted therapy than non-carriers (Table 3), which is similar to what was found in a previous study using international data (29).*~~*"*

Comment 3: Additionally, this study and manuscript will contribute most meaningfully to the scientific community if the results presented aid in the understanding of both patients and physicians about the risks of contralateral breast cancer in these mutation carriers so that this information can be used to help patients make more tailored decisions about the benefit of contralateral prophylactic mastectomy once diagnosed with a breast cancer. Stating that there is an increased risk of contralateral breast cancer risk, without given specifics with cumulative incidence of contralateral breast cancer risk at 5, 10, 15 years is less helpful. At this point, the follow-up period may not be long enough to provide this data. However, given that the study has a cohort of carriers, and non-carriers, my suggestion would be do present the increase in contralateral breast cancer using Hazard ratios. Presenting a table showing this would be helpful.

Response 3: We agree that a cumulative incidence of contralateral breast cancer risk at 5, 10 and 15 years would be more helpful. However, in our opinion, cumulative incidences or hazard ratios comparing contralateral breast cancer risk in heterozygotes (n=856, of whom 207 develop contralateral breast cancer) and familial non-carriers (n=64, of whom 10 develop contralateral breast cancer) might not be useful as the numbers of the latter group are too small. A longer follow-up and larger group of non-carriers would be more useful and will be available in the nearby future.
We have instead added the percentage of contralateral breast cancer cases over 5, 10, and 15 years.

Comment 4: Additionally, the authors fail to include as reference the most recent and high impact manuscript from the CARRIERS study which examined contralateral breast cancer risk(Yadav S, Boddicker NJ, Na J, Polley EC, Hu C, Hart SN, Gnanaolivu RD, Larson N, Holtegaard S, Huang H, Dunn CA, Teras LR, Patel AV, Lacey JV, Neuhausen SL, Martinez E, Haiman C, Chen F, Ruddy KJ, Olson JE, John EM, Kurian AW, Sandler DP, O'Brien KM, Taylor JA, Weinberg CR, Anton-Culver H, Ziogas A, Zirpoli G, Goldgar DE, Palmer JR, Domchek SM, Weitzel JN, Nathanson KL, Kraft P, Couch FJ. Contralateral Breast Cancer Risk Among Carriers of Germline Pathogenic Variants in ATM, BRCA1, BRCA2, CHEK2, and PALB2. J Clin Oncol. 2023 Mar 20;41(9):1703-1713. doi: 10.1200/JCO.22.01239. Epub 2023 Jan 9. PMID: 36623243; PMCID: PMC10022863) I would suggest the authors read this manuscript if they have not and also include it as reference to ensure references are up to date. As a point of interest, given that this study has a larger cohort of CHEK2 patients than the CARRIERS study, it would be interesting if they conducted an exploratory analysis examining the risk of contralateral breast cancer in the CHEK2 mutation carriers by ER status and menopausal status as differences in the risk of contralateral breast cancer were found in the CARRIERS study based off these factors. At this point, we still have limited understanding about CHEK2-associated breast cancer, particularly about the risk of contralateral breast cancer in these individuals.

Response 4: Thank you for mentioning this manuscript. We agree that this research is very relevant and the reference has been added in the introduction. One of the aims of this cohort is to (also) study

the contralateral breast cancer risk, however, at the moment we do not have enough power to study contralateral breast cancer risk while taking the moment of genetic testing into account (left-truncated analysis). More events and a longer follow-up are needed to study this in more detail, also allowing to take the ER-status and menopausal status into account. This has also been clarified in future plans.

Introduction (page 4, line 89-91):
"Furthermore, after the primary breast cancer diagnosis, carriers (both heterozygous and homozygous) more often develop contralateral breast cancer (16, 20-22) and tend to have a worse survival than non-CHEK2 carriers (16-18, 23)."

Future plans (page 10, line 287-290):
"This will result in longer follow-up time for known breast cancer cases and identify new breast cancer patients ~~enabling us~~ increasing power to prospectively assess (contralateral) breast cancer risk, also allowing for further stratification based on ER-status and menopausal status. Furthermore, ~~and~~ this would enable us to study prognosis after breast cancer occurrence in the least biased way."

Comment 5: This cohort and study can provide valuable knowledge to improve risk prediction and cancer care in CHEK2 mutation carriers for years to come. However, further details related to risks associated with these mutations with regards to contralateral breast cancer will increase the impact of this manuscript to the scientific community. Additionally, as the authors ultimately wish to use this cohort to expand our knowledge potentially of additionally cancer risks, I would encourage them to expand their cohort to include men, especially with emerging data related to elevated prostate cancer risk.

Response 5: We agree that expanding this cohort to include men is important. We aim to do this in the future, so we can study cancer risk in men as well. This has been clarified in the discussion.

Future plans (page 10, line 292-294):
"In addition, we aim to expand the cohort, also to include tested males and tested relatives from families where other (likely) pathogenic CHEK2 variants are found. Including these families in this cohort would allow us to study risks for these families as well and compare them with CHEK2 c.1100delC families."

In addition, we have studied cancer risk in CHEK2 c.1100delC families. In the pedigrees, there was too limited information on cancer risk in men, possibly as a result of the study design as we included women with breast cancer and their families. However, we still found an increased risk of breast cancer and colorectal cancer, but not prostate cancer. We have added information on this study in the findings to date as well.

Findings to date (page 8, line 238-242):
"Using pedigree information from 609 CHEK2 c.1100delC families, we studied whether families are at risk of other cancers in addition to the breast (31). In this study, we found an increased risk of colorectal cancer and haematological cancers for women from CHEK2 c.1100delC families. Even though there

seems to be an underreporting of cancer in the male relatives in this study, we also found an increased risk of breast cancer and colorectal cancer in men."

Reviewer #2:
Thanks to the authors for their excellent work. I only have two comments:
Comment 1: Has the population included in the study been tested for mutations other than the CHEK2 c.1100delC variant to exclude a concomitant mutation?

Response 1: Thank you for your comment. As far as possible, we excluded concomitant variants. However, as women are diagnostically tested, the extent of genetic testing in women differs. Therefore, we are unsure whether other variants might be present in these women. This has been clarified in the cohort description.

Cohort description; selection, invitation and response (page 5, line 138-142):
"As the selection was based on CHEK2 c.1100delC status only, it might be possible that women from families with other pathogenic variants in addition to the CHEK2 c.1100delC are included in this study. However, when this information was available, these women were excluded. Overall, the likelihood of compound CHEK2 heterozygosity or the combination of CHEK2 c.1100delC and other pathogenic variants in one of the breast cancer-associated genes is small."

Comment 2: Table 2 shows the available information on the characteristics of the patients. Was any univariate/multivariate analysis performed to evaluate the role in the association with the CHEK2 mutation? I am aware that some habits are already known as potential risk factors for breast cancer (smoking, alcohol consumption), but it might be interesting to comment on them.

Response 2: We agree that further analyses regarding associations with *CHEK2* c.1100delC would be interesting. At the moment, no univariate/multivariate analyses were performed. However, this might be studied in the future. We aim to increase follow-up time resulting in more events, and possibly also send out additional questionnaires to study differences in risk factors over time. However, based on current literature, we do not expect that there would be other risk factors for breast cancer in *CHEK2* c.1100delC than in other hormone sensitive breast cancer patients.

VERSION 2 - REVIEW

**Reviewer**	**1**
**Name**	**Whitaker , Kristen**
**Affiliation**	**MedStar Health**
**Date**	**26-Sep-2024**
**COI**	**None**

This is meaningful research and with continued follow up of the cohort will provide important knowledge to allow providers to more effectively counsel patients and improve cancer prevention in these mutation carriers.

Suggestions from original review have been appropriately addressed and no further revisions recommended.

Excellent manuscript.

**Reviewer**	**2**
**Name**	**Moscetti , Luca**
**Affiliation**	**Universitaria di Modena, Oncology**
**Date**	**28-Aug-2024**
**COI**	**none**

I would like to thanks the authors for the response, the article can be accepted.
